# Colorectal Carcinoma Metastatic to the Breast Mimicking Inflammatory Breast Cancer With Mismatch Repair Status Discordant From a Prior Endometrial Primary: A Case Report and a Review of the Literature

**DOI:** 10.7759/cureus.115369

**Published:** 2026-08-28

**Authors:** Morgan L Walters, Andres Figueroa Quast

**Affiliations:** 1 Internal Medicine, Mercy Hospital St. Louis, St. Louis, USA

**Keywords:** breast cancer, extramammary breast mass, metastatic colo-rectal cancer, pms2, uterine cancer

## Abstract

Colorectal carcinoma (CRC) metastatic to the breast is exceedingly rare, with 46 cases compiled in a 2019 literature review and additional isolated reports since. The diagnostic challenge is substantially amplified in patients with a prior breast cancer history, in whom new breast lesions are reflexively attributed to recurrence rather than extramammary metastasis.

A 71-year-old woman with a significant family history of malignancy presented with invasive lobular carcinoma of the left breast, treated with lumpectomy and hormonal therapy. Four months later, she was diagnosed with stage I endometrioid adenocarcinoma of the uterus; immunohistochemistry of the hysterectomy specimen revealed isolated PMS2 loss with preserved MLH1, MSH2, and MSH6 expression, and comprehensive germline testing of 35 hereditary cancer predisposition genes was negative, establishing a Lynch-like phenotype in that tumor. Approximately four years after her breast cancer diagnosis, she developed a maculopapular rash with skin thickening of the left breast, without a discrete mass; the clinical and mammographic presentation was concerning for inflammatory breast carcinoma. Punch biopsy demonstrated poorly differentiated adenocarcinoma with signet ring cell features, immunohistochemically positive for SATB2, CDX2, CK20, and CEA and negative for CK7, ER, PR, HER2, and GATA3, confirming colorectal origin and excluding breast cancer recurrence. Mismatch repair immunohistochemistry on the same specimen demonstrated retained expression of all four proteins, discordant with the endometrial primary and concordant with microsatellite instability-stable status and a tumor mutational burden of 3.7 mutations/Mb. Next-generation sequencing identified a TP53 splice region loss-of-function variant with RAS and BRAF wild-type status. Positron emission tomography revealed stage IV disease with hepatic metastasis, and colonoscopy identified synchronous cecal and recto-sigmoid masses. She was treated with four lines of systemic therapy and palliative radiation to the breast, and died approximately 20 months after the metastatic diagnosis.

This case illustrates two points. First, any new breast lesion in a patient with prior malignancy requires histopathologic confirmation with comprehensive immunohistochemical analysis, as neither recurrence nor a primary breast process can be assumed. Second, mismatch repair status is a property of the individual tumor rather than of the patient; in patients with multiple primaries, each tumor requires independent testing, and molecular findings from one cannot be extrapolated to another.

## Introduction

Colorectal cancer (CRC) is the second leading cause of cancer-related death in the United States, with an estimated 158,850 new cases expected in 2026 [1]. CRC most frequently metastasizes to the liver, reflecting portal venous drainage of the colon directly to the hepatic sinusoids. Metastasis to the breast is extremely rare; a 2019 literature review compiled 46 reported cases [2], and a series spanning 1907 to 1999 found that only 0.43% of breast malignancies originated outside the breast [3]. Because extramammary metastasis to the breast is so uncommon, a new breast lesion in a patient with a prior breast primary is almost reflexively attributed to recurrence, an anchoring bias that can be resolved only by histopathologic and immunohistochemical confirmation.

The mismatch repair (MMR) system, comprising MLH1, MSH2, MSH6, and PMS2, corrects base-base mismatches and insertion-deletion loops arising during DNA replication. Loss of MMR function produces microsatellite instability (MSI), a hypermutable phenotype identified either by polymerase chain reaction-based testing of microsatellite loci or inferred from loss of MMR protein expression on immunohistochemistry [4]. MSI-high status is clinically actionable, establishing eligibility for first-line immune checkpoint inhibition in metastatic CRC [5]. MMR protein loss on tumor immunohistochemistry also raises the possibility of Lynch syndrome, an autosomal dominant cancer predisposition caused by germline MMR gene mutation. When tumor MMR deficiency is demonstrated but comprehensive germline testing identifies no pathogenic variant, the phenotype is designated "Lynch-like", a category in which biallelic somatic inactivation of MMR genes accounts for a substantial proportion of cases [6]. MMR protein expression is a property of the individual tumor rather than of the patient, and inter-tumoral discordance has been documented among patients with more than one primary gastrointestinal carcinoma [7].

Here we present a woman who developed three metachronous primary malignancies: invasive lobular carcinoma of the breast, endometrioid adenocarcinoma of the uterus diagnosed four months later, and colorectal carcinoma. Four years after her breast cancer diagnosis, a new lesion in the treated breast proved to be metastatic colorectal carcinoma rather than recurrence. MMR immunohistochemistry performed on both the endometrial and colorectal specimens yielded discordant results, and comprehensive germline testing of 35 hereditary cancer predisposition genes was negative.

## Case presentation

**Figure 1:**
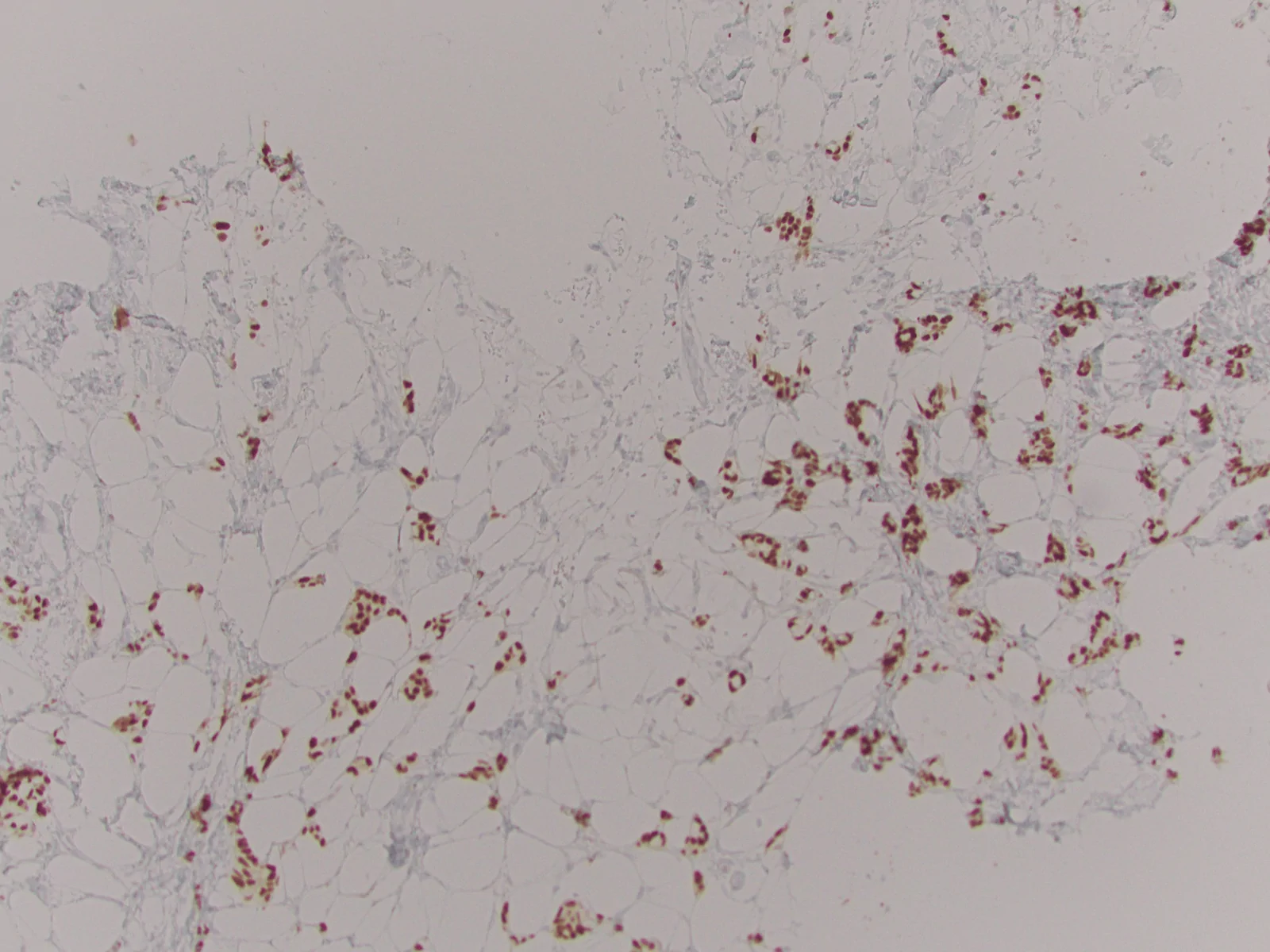
Left breast biopsy which confirmed ER-positive breast cancer (immunohistochemistry; 10x) Image credit to Dr. Jena Weimholt, Medical Director of Cytology at Mercy Hospital St. Louis. ER - estrogen receptor

 A 71-year-old woman with a significant family history of malignancy, including a mother with colon cancer and a sister with bilateral breast cancer and uterine cancer (Table 1), presented following a routine screening mammogram that showed an abnormality in the left breast at the 2 o'clock position. This ultimately led to a biopsy that confirmed invasive lobular carcinoma, which was estrogen receptor (ER)-positive and human epidermal growth factor receptor 2 (HER2)-negative (Figure 1). She underwent a lumpectomy, which had negative margins as well as a negative sentinel lymph node. Her Oncotype DX testing was used to risk stratify and indicated adjuvant chemotherapy would not be beneficial. Radiation was offered, but the patient deferred.

**Table 1:** Family history summary Ages provided represent age at diagnosis for each malignancy. ER - estrogen receptor; HER2 - human epidermal growth factor receptor 2; PMS2 - PMS1 homolog 2

Generation	Individual	Sex	Malignancy	Age at diagnosis	Relevance
I	Mother	F	Colon cancer	50	—
II	Sister	F	Bilateral breast cancer	55	—
II	Sister	F	Uterine cancer	Not reported	—
II	Patient	F	Invasive lobular carcinoma	71	ER+/HER2-
II	Patient	F	Endometrial carcinoma	71	Stage I; isolated PMS2 loss; Lynch-like phenotype
II	Patient	F	Colorectal adenocarcinoma	75	Metastatic; TP53 mutation; mismatch repair proficient

**Figure 2:**
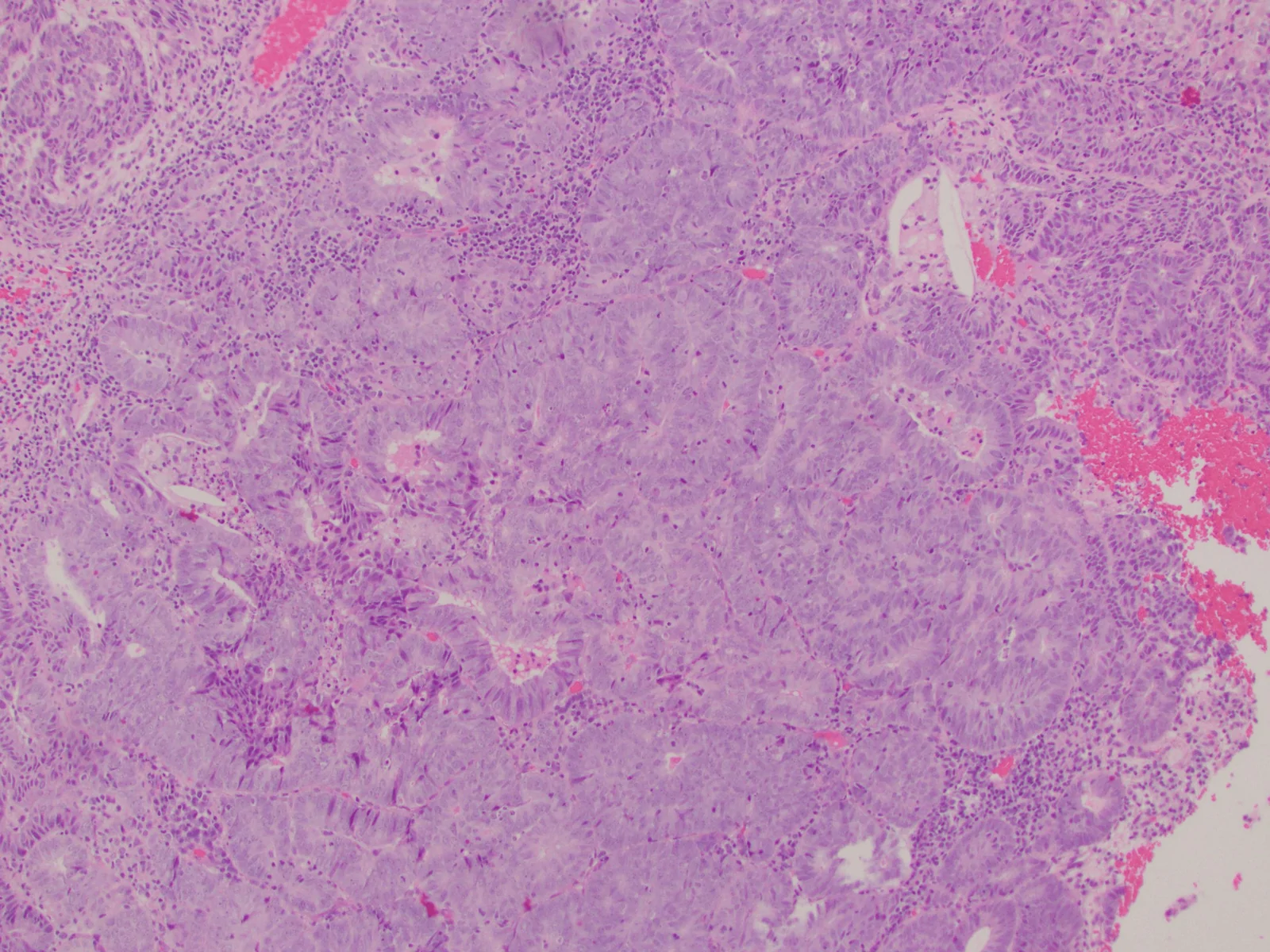
Endometrial biopsy confirming stage I endometrioid adenocarcinoma of the uterus (hematoxylin and eosin; 20x) Image credit to Dr. Jena Weimholt, Medical Director of Cytology at Mercy Hospital St. Louis.

Four months after diagnosis, she underwent a pelvic ultrasound for painless vaginal spotting. Imaging was pertinent for a 16.9 mm endometrial stripe. Therefore, an in-office endometrial biopsy was performed and ultimately led to the diagnosis of stage I endometrioid adenocarcinoma of the uterus (Figure 2). She was agreeable to undergoing total hysterectomy and bilateral salpingo-oophorectomy. Immunohistochemistry of the hysterectomy specimen revealed isolated PMS2 loss with preserved expression of MLH1, MSH2, and MSH6 (Table 2), raising concern for an underlying hereditary cancer predisposition. The tumor was estrogen receptor and progesterone receptor positive. She was initiated on anastrozole daily and, after extensive discussion, declined radiation for her uterine cancer as well. Given this finding and her significant family history of malignancy, she was referred for comprehensive germline genetic testing, which was negative for deleterious mutations in all 35 genes tested, including those responsible for Lynch syndrome and hereditary breast and ovarian cancer syndrome, as well as most other common hereditary cancer syndromes.

**Table 2:** Mismatch repair protein immunohistochemistry by specimen Immunohistochemistry for MLH1, MSH2, MSH6, and PMS2 was performed separately on each specimen. The left breast punch biopsy contained metastatic colorectal carcinoma; results in that column therefore reflect the colorectal tumor. Microsatellite instability testing on the breast punch biopsy was stable, with a tumor mutational burden of 3.7 mutations/Mb. MMR, mismatch repair.

MMR protein	Endometrioid adenocarcinoma (uterus, hysterectomy)	Metastatic colorectal carcinoma (left breast punch biopsy)
MLH1	Retained	Retained
MSH2	Retained	Retained
MSH6	Retained	Retained
PMS2	Loss of expression	Retained
Interpretation	Mismatch repair deficient	Mismatch repair proficient

**Figure 3:**
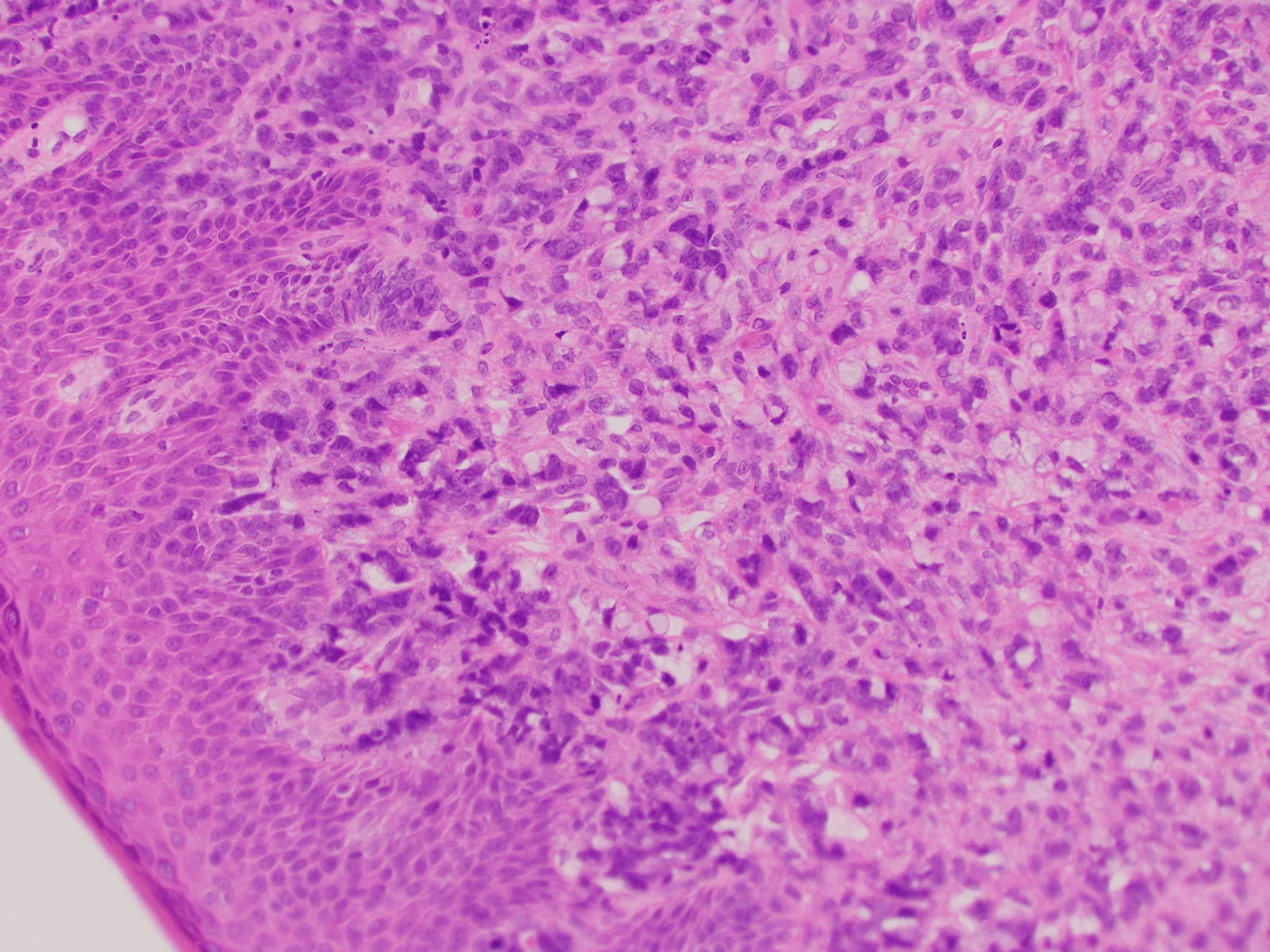
Punch biopsy of left breast showing signet ring cells, supporting a gastrointestinal origin (hematoxylin and eosin; 20x) Image credit to Dr. Jena Weimholt, Medical Director of Cytology at Mercy Hospital St. Louis.

**Figure 4:**
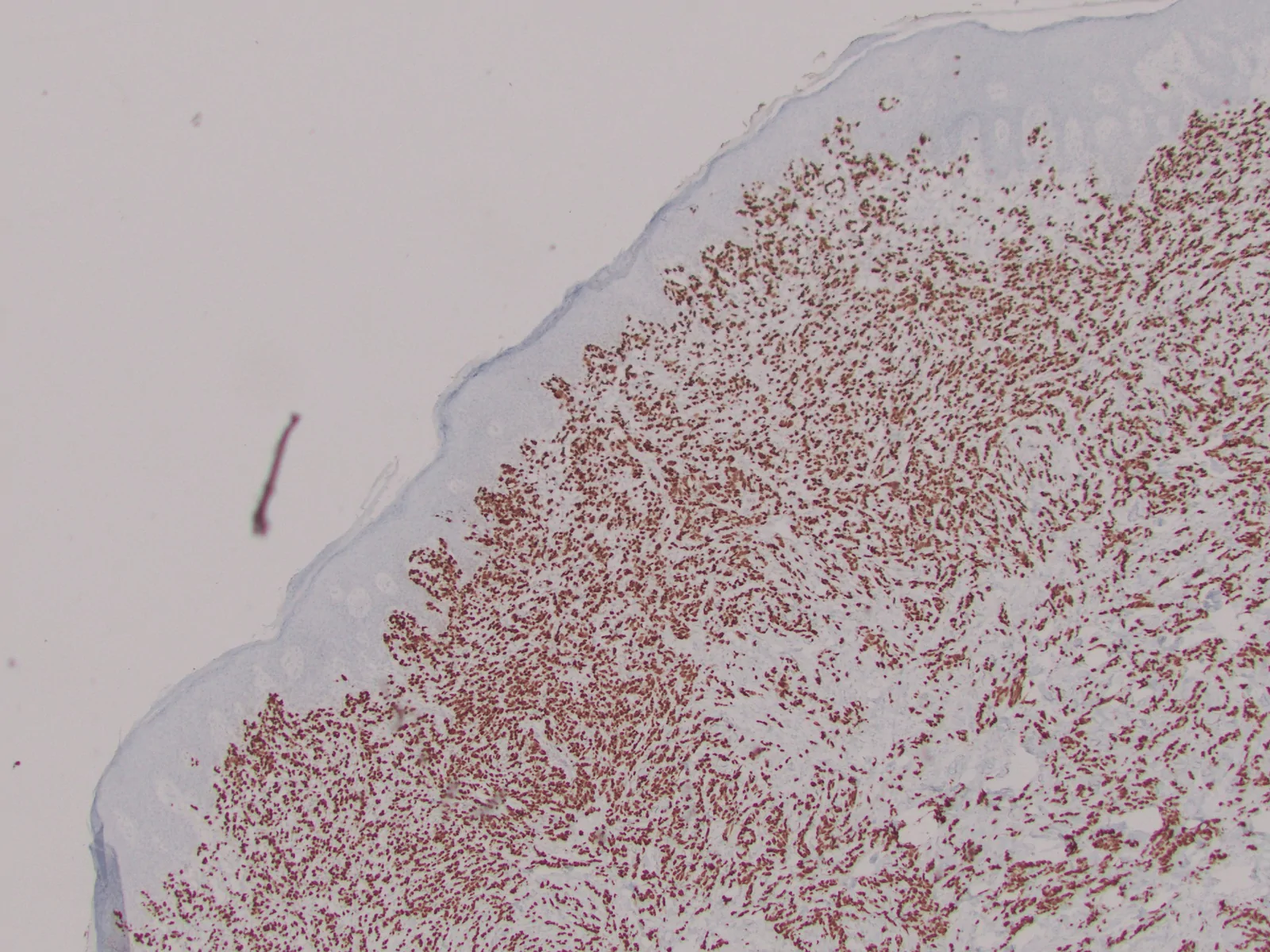
Breast punch biopsy positive for CDX2 (immunochistochemistry; 10x) Image credit to Dr. Jena Weimholt, Medical Director of Cytology at Mercy Hospital St. Louis.

**Figure 5:**
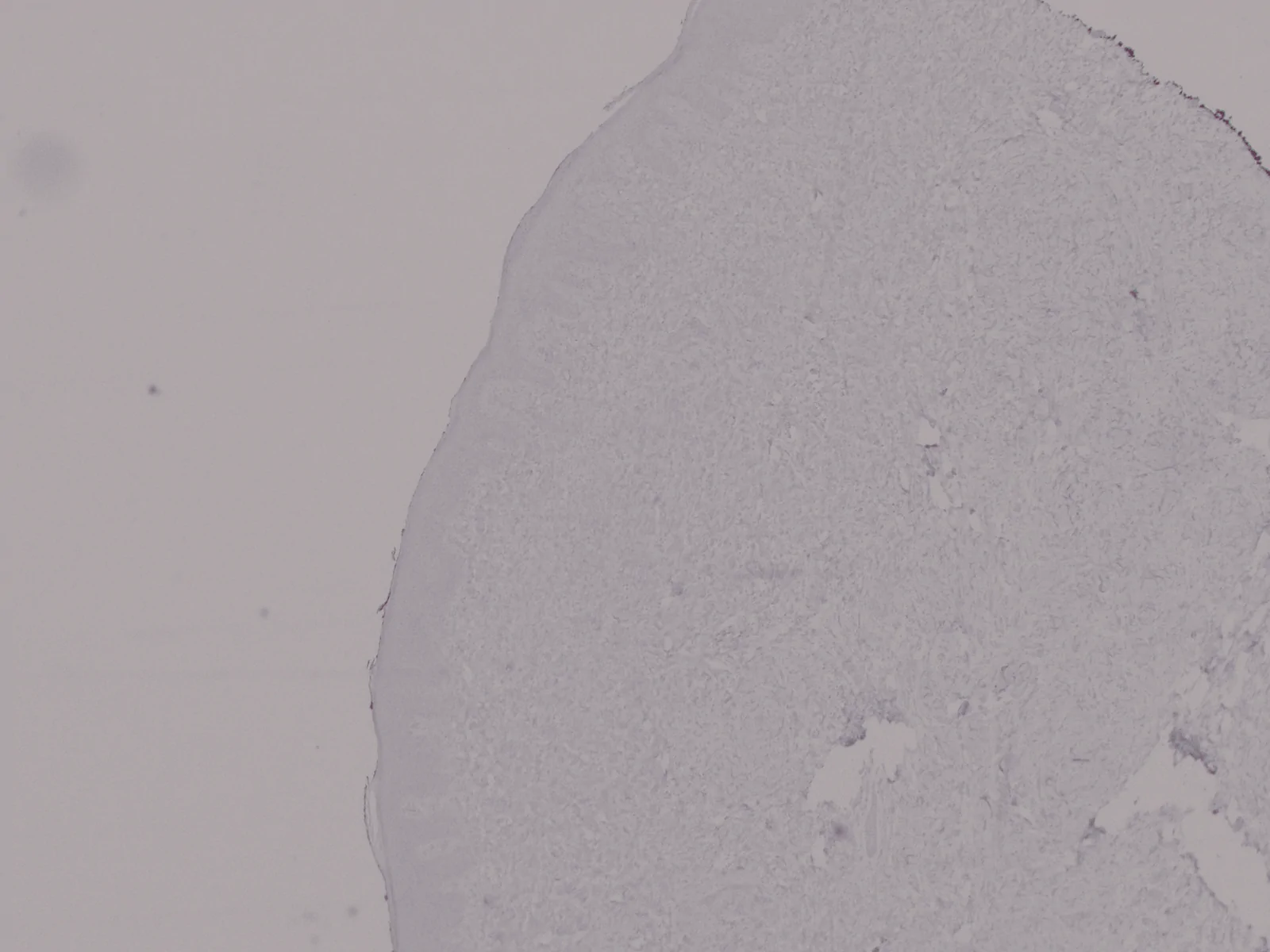
Punch biopsy of left breast negative for GATA3 (immunohistochemistry; 10x) Image credit to Dr. Jena Weimholt, Medical Director of Cytology at Mercy Hospital St. Louis.

She did well with routine follow-up for several years until approximately four years after initial breast cancer diagnosis, at age 75, she presented with a maculopapular rash involving the lateral aspect of the left breast, with extension to the areola. Physical examination revealed minimal tenderness to palpation and no palpable underlying breast mass. Mammography revealed BIRADS category 4 (suspicious) findings, including progressive increase in left breast density, progressive skin thickening, and rash appearance, without a discrete mass. The radiologic and clinical presentation were highly concerning for inflammatory breast cancer. Given this clinical-radiologic correlation, punch biopsy of the skin was performed to evaluate the breast lesion. Histopathology demonstrated poorly differentiated adenocarcinoma with signet ring cell features, morphologically inconsistent with her known invasive lobular carcinoma (Figure 3). Immunohistochemical analysis was pertinent for positivity of SATB2, CDX2, CK20, and CEA (Figure 4). Immunohistochemistry analysis was negative for CK7, ER, PR, HER2, and GATA3 (Figure 5), confirming colorectal origin and excluding breast cancer recurrence. Mismatch repair immunohistochemistry performed on the same specimen demonstrated retained expression of MLH1, MSH2, MSH6, and PMS2, indicating a mismatch repair proficient tumor and contrasting with the isolated PMS2 loss seen in the endometrial primary (Table 2). Next-generation sequencing of the diagnostic breast punch biopsy (Tempus xT, 648-gene DNA panel, tumor-normal matched analysis) identified a TP53 splice region variant (c.376-1G>C, loss of function, variant allele fraction 48.7%). KRAS, NRAS, and BRAF were wild type, and HER2 was negative, supporting subsequent EGFR therapy. Microsatellite instability status was stable, with a tumor mutational burden of 3.7 mutations/Mb, precluding first-line pembrolizumab. PD-L1 (22C3) combined positive score was 52.

**Figure 6:**
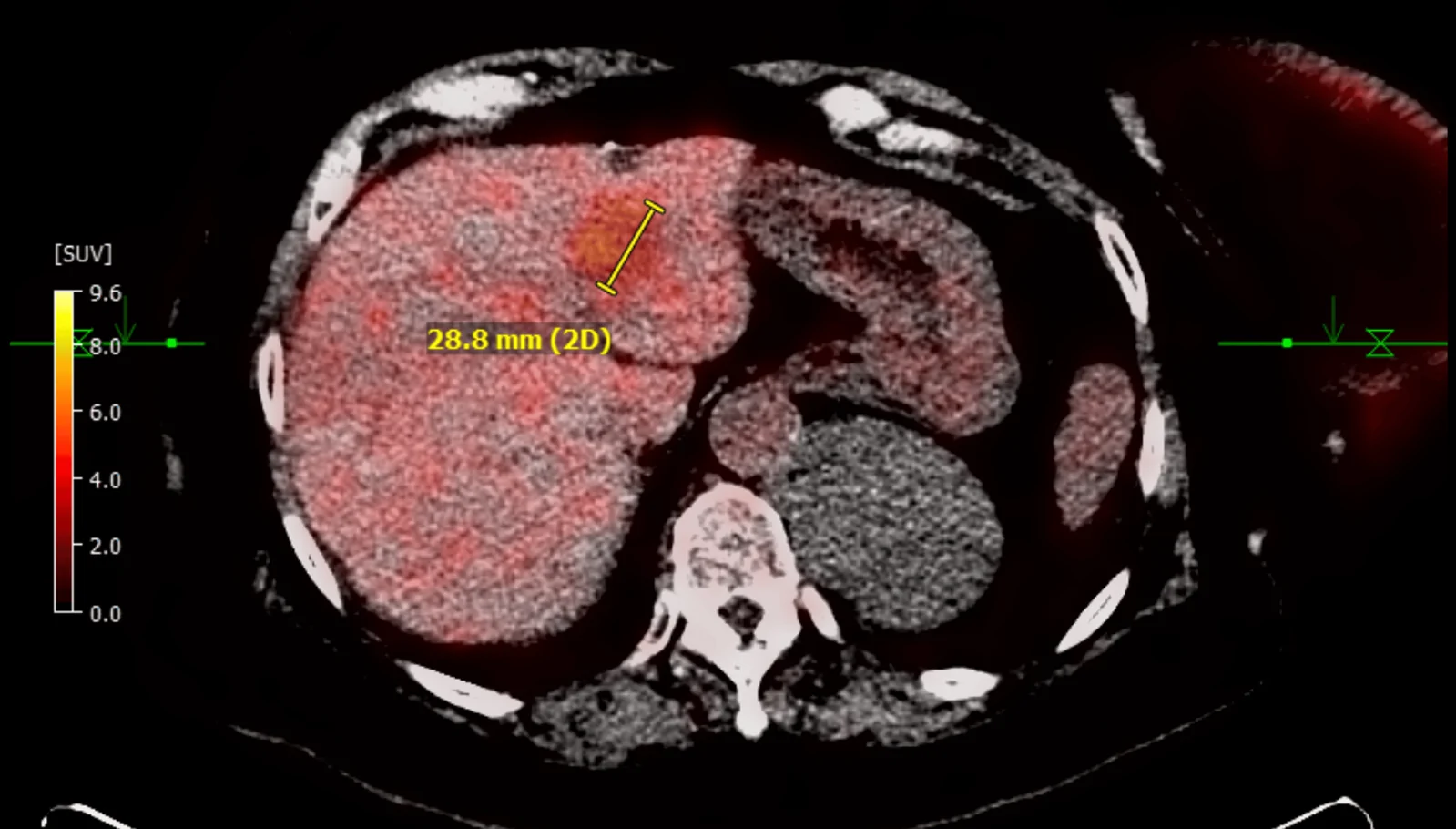
PET scan showed an FDG-avid lesion within the liver measured at 28.8 mm

Positron emission tomography (PET) demonstrated fluorodeoxyglucose (FDG)-avid diffuse involvement of the left breast in an inflammatory-appearing distribution, with a solitary hypermetabolic metastasis in the left hepatic lobe (Figure 6). In light of the punch biopsy findings, these were interpreted as stage IV metastatic colorectal carcinoma involving the breast rather than primary inflammatory breast carcinoma. Subsequent colonoscopy revealed an infiltrative cecal mass and a fungating partially obstructing mass within the recto-sigmoid colon. Upper endoscopy was unremarkable. This new diagnosis prompted the initiation of first-line FOLFIRINOX (folinic acid, fluorouracil, irinotecan, and oxaliplatin) every two weeks. She unfortunately had severe cytopenias due to therapy, necessitating dose reductions and multiple cycle delays. Following cycle five, computed tomography (CT) scan of chest, abdomen, and pelvis showed relatively stable disease along with down-trending CA19-9 (74 to 37 U/mL) and CEA (39.6 to <2 ng/mL). On cycle eight, treatment was switched from FOLFIRINOX to FOLFOX (folinic acid, fluorouracil, and oxaliplatin), given her consistently low blood counts and frequent cycle delays; this represented a toxicity-driven modification within first-line therapy rather than a change of line, as there was no evidence of disease progression at that time. 

**Figure 7:**
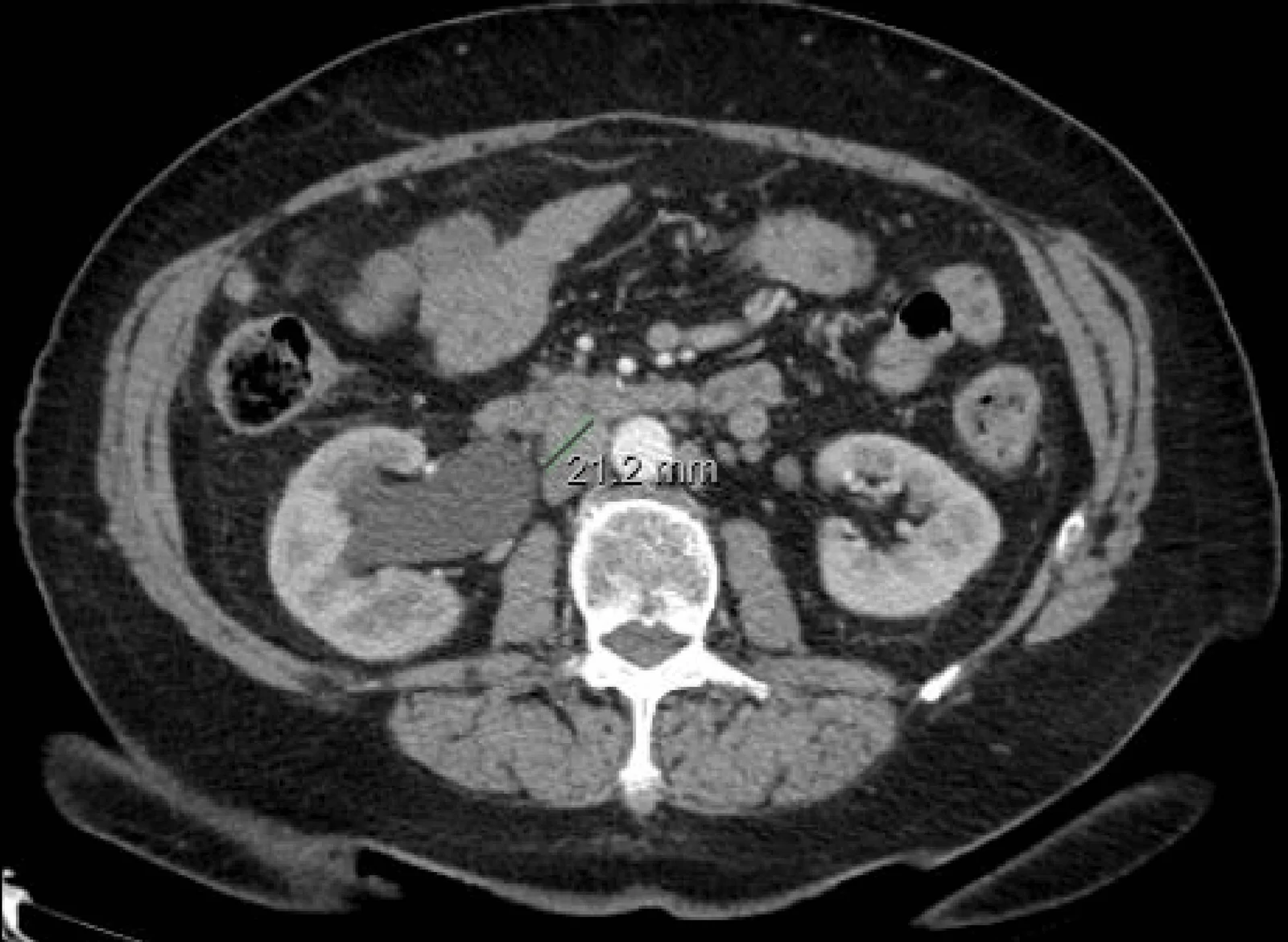
Restaging CT after with one cycle of maintenance 5fu-lucovorin demonstrated increased retroperitoneal lymph nodes, measured at 21.2 mm

**Figure 8:**
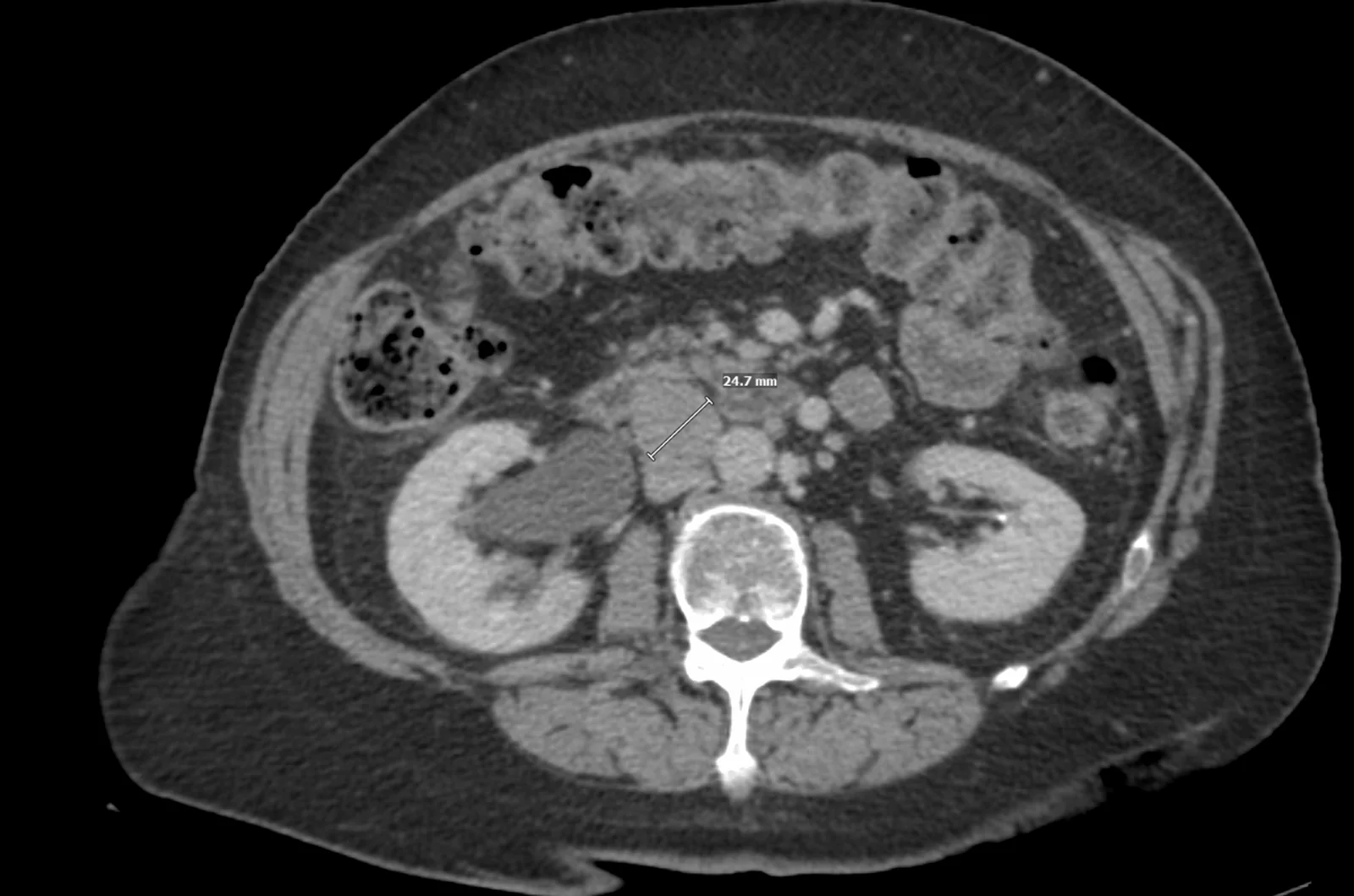
Restaging CT after 6 cycles of panitumumab and irinotecan again showed increased retroperitoneal lymphadenopathy (to 24.7 mm)

Following 12 cycles, she began maintenance with one cycle of 5-FU/leucovorin, continuing within first-line therapy. Restaging CT imaging demonstrated increasing retroperitoneal lymph nodes (Figure 7). The patient was discussed during a multidisciplinary tumor board, and a decision was made to transition to second-line panitumumab and irinotecan every two weeks. She completed six cycles of this regimen before restaging CT again showed progression (Figure 8), although a relatively stable CEA level of 3.6 ng/mL. She then began third-line treatment with bevacizumab and trifluridine/tipiracil. She tolerated this regimen very poorly, with a decline in functional status. She eventually progressed on this treatment as well and was started on fourth-line treatment with fruquintinib, at which time her CEA was 17.1 ng/mL. Due to the ongoing pain and discomfort of her left breast, she underwent palliative radiation; an ultra-hypofractionated course in five fractions, which improved her symptoms for a brief time. Despite all treatments, she developed severe abdominal pain due to a large bowel obstruction secondary to a mass. Ultimately, the decision was made to transition to hospice, and she passed away shortly after, approximately 20 months after the diagnosis of metastatic colorectal carcinoma. At this time, her final CEA level was 93.9 ng/mL.

## Discussion

Colorectal carcinoma metastatic to the breast is exceedingly rare. A 2019 literature review compiled 46 reported cases [2]; additional isolated cases have been published subsequently [8,9], though no updated cumulative tally exists. In a series spanning 1907 to 1999, only 0.43% of breast malignancies were metastatic deposits from a non-mammary primary [3]. The diagnostic challenge is substantially compounded in patients with a prior breast cancer history, in whom new breast lesions are overwhelmingly attributed to recurrence rather than extramammary metastasis, a form of anchoring bias with significant clinical consequences.

In our patient, two features could plausibly have anchored the diagnosis. Her history of ipsilateral invasive lobular carcinoma made recurrence the intuitive explanation, and the clinical and mammographic presentation, a maculopapular rash with skin thickening and increasing breast density in the absence of a discrete mass, was independently concerning for inflammatory breast carcinoma. Both are reasonable initial impressions, and both were incorrect. Diagnosis was established by punch biopsy demonstrating poorly differentiated adenocarcinoma with signet ring cell features, morphologically inconsistent with her known lobular carcinoma. As shown in Table 3, immunohistochemistry was decisive: positivity for SATB2, CDX2, CK20, and CEA with negativity for CK7, ER, PR, HER2, and GATA3 is a profile highly sensitive and specific for colorectal origin and effectively excludes recurrent breast malignancy [10]. This case reinforces that any new breast lesion in a patient with prior malignancy warrants histopathologic confirmation with comprehensive immunohistochemical analysis, regardless of how strongly recurrence or a primary breast process is suspected.

**Table 3:** Immunohistochemical panel of the breast punch biopsy demonstrating a colorectal, rather than breast, origin of the metastatic lesion.

Marker	Result	Interpretation
SATB2	Positive	Supports colorectal origin
CDX2	Positive	Supports colorectal origin
CK20	Positive	Supports colorectal origin
CEA	Positive	Supports colorectal origin
CK7	Negative	Argues against breast origin
ER	Negative	Argues against breast origin (patient's prior primary was ER-positive)
PR	Negative	Argues against breast origin
HER2	Negative	Argues against breast origin
GATA3	Negative	Argues against breast origin

Mismatch repair immunohistochemistry was performed on both of this patient's non-breast primaries and yielded discordant results. The endometrial carcinoma demonstrated isolated PMS2 loss with retained MLH1, MSH2, and MSH6, a pattern that argues against MLH1 promoter hypermethylation and, with negative germline testing across 35 hereditary cancer predisposition genes, meets criteria for a Lynch-like phenotype [6]. The colorectal carcinoma, sampled through the breast punch biopsy, demonstrated retained expression of all four mismatch repair proteins, concordant with MSI-stable status and a tumor mutational burden of 3.7 mutations/Mb on the same specimen. The colorectal tumor is therefore mismatch repair proficient, and the Lynch-like designation pertains to the endometrial primary alone.

This discordance carries a practical implication. Isolated PMS2 loss on tumor immunohistochemistry predicts MSI-high status, and had the endometrial result been extrapolated to the colorectal carcinoma, first-line pembrolizumab would have appeared to be a reasonable consideration on the basis of KEYNOTE-177 [5]. Independent testing of the colorectal specimen established MSI-stable status and correctly excluded that option. Current NCCN guidelines recommend universal mismatch repair and microsatellite instability testing on all newly diagnosed colorectal carcinoma specimens regardless of prior results [11]; this case illustrates that recommendation functioning as intended, and specifically supports testing each primary independently in patients with more than one malignancy [7]. Mismatch repair status is a property of the individual tumor, not of the patient.

The TP53 splice region loss-of-function variant identified on sequencing is not distinguishing in this context, as TP53 mutations occur in approximately 40-60% of colorectal carcinomas [12]. Her trajectory, with rapid progression through four lines of systemic therapy and death approximately 20 months after the metastatic diagnosis, is broadly consistent with the reported mean survival of 14.9 months for colorectal carcinoma metastatic to the breast [2].

This report is limited by its single-case design; the molecular observations are descriptive and hypothesis-generating. Comprehensive sequencing was performed only on the colorectal carcinoma, and MLH1 promoter methylation testing was not performed on the endometrial specimen, so the mechanism underlying the isolated PMS2 loss was not established.

## Conclusions

This case describes colorectal carcinoma metastatic to the breast in a woman with three metachronous primary malignancies, presenting as a cutaneous eruption of a previously treated breast that was clinically and radiographically concerning for both recurrence and inflammatory breast carcinoma. The principal lesson is diagnostic: when a new breast lesion develops in a patient with prior breast cancer, recurrence is the instinctive interpretation, and histopathologic confirmation with a comprehensive immunohistochemical panel remains essential regardless of how compelling that interpretation appears. In this patient, immunohistochemistry alone distinguished metastatic colorectal carcinoma from recurrent lobular carcinoma and redirected management entirely.

Mismatch repair immunohistochemistry performed on both non-breast primaries yielded discordant results, with isolated PMS2 loss in the endometrial carcinoma and retained expression of all four proteins in the colorectal carcinoma. This observation is descriptive and derived from a single case, but it illustrates that mismatch repair status is a property of the individual tumor rather than of the patient, and supports independent testing of each primary in patients with more than one malignancy rather than extrapolation between them.
